# Aerogels for Carbon Dioxide Capture: Classification Design, Preparation Strategies, and Capture Scenarios

**DOI:** 10.3390/gels12090797

**Published:** 2026-09-01

**Authors:** Yang Yang, Yu Mao, Chao Sun, Jingna Jia, Xinyu Li

**Affiliations:** 1 College of Engineering, Materials and Chemical Engineering, Yanbian University, Yanji 133002, China; 2025050044@ybu.edu.cn; 2 Department of Polymer Materials & Engineering, College of Engineering, Yanbian University, Yanji 133002, China; 1244022505@ybu.edu.cn; 3 Department of Physics, Jilin University, Changchun 130012, China; sunc344@jlu.edu.cn; 4 Department of Chemistry, College of Science, Yanbian University, Yanji 133002, China

**Keywords:** aerogels, carbon dioxide capture, composite aerogels, adsorption

## Abstract

The global atmospheric CO_2_ concentration continues to rise, leading to increasingly severe greenhouse effects, ocean acidification, and extreme climate events. Therefore, the development of efficient CO_2_ capture materials is urgently needed. Aerogels, a class of three-dimensional nanoporous solid materials formed by the crosslinking of nanoparticles or polymer molecular chains via the sol–gel process, exhibit outstanding advantages in CO_2_ capture due to their high specific surface area, tunable nanopores, and abundant surface functionalizable sites. This paper systematically summarizes the preparation methods, including supercritical drying, ambient pressure drying, and freeze drying, reviews the classification and design strategies of aerogel materials, and analyzes the application status of aerogels in scenarios ranging from direct air capture, post-combustion flue gas capture, and natural gas purification to carbon sequestration. Finally, future development trends are prospected, aiming to provide a reference for the design and large-scale application of high-performance aerogel-based CO_2_ adsorbents.

## 1. Introduction

The continuous increase in atmospheric CO_2_ concentration, along with the resulting global warming, ocean acidification, and frequent extreme climate events, poses a direct threat to the sustainable development of human society, making carbon neutrality a global consensus [1]. Among the numerous carbon capture technologies, solid adsorption has attracted widespread attention due to its advantages such as simple operation, low regeneration energy consumption, and non-corrosiveness [2,3]. Currently, the main solid CO_2_ adsorbents include amine-modified mesoporous silica [4], activated carbon [5], zeolites [6,7], and metal–organic frameworks (MOFs) [8]. However, amine-modified mesoporous silica suffers from amine leaching and pore blockage; activated carbon, despite its high specific surface area, exhibits a relatively low adsorption selectivity difference between CO_2_ and N_2_; zeolites show a sharp decline in adsorption capacity under humid conditions; and MOFs, although structurally tunable, face insufficient hydrothermal stability and high synthesis costs. A comparison of these materials with aerogels is presented in Table 1. Therefore, there is an urgent need to develop novel CO_2_ adsorbent materials that combine a high specific surface area, tunable pore structures, abundant surface chemical sites, and excellent cycling stability.

Aerogels are a class of three-dimensional porous network solid materials formed by the crosslinking of nanoparticles or polymer molecular chains via sol–gel processes. They exhibit densities as low as 3–500 mg·cm^−3^, specific surface areas up to 500–3000 m^2^·g^−1^, and porosities ranging from 80% to 99.8% [9]. Since Kistler first prepared SiO_2_ aerogels by supercritical drying in 1931, the material systems of aerogels have expanded from single-component silica to carbon materials, polymers, biomass, and their composites [10]. Benefiting from their tunable nanoporous architectures, abundant surface functional groups, and low skeletal density, aerogels meet the requirements for high-capacity and rapid CO_2_ adsorption, thus demonstrating unique structural advantages in the field of CO_2_ capture [11]. In recent years, the number of research papers on CO_2_ adsorption of aerogels has grown rapidly, covering various strategies for preparing aerogels such as organic amine modification and MOF composites [12]. However, the current field has a large body of literature, but they are scattered from each other, lacking theoretical integration and systematic review papers.

Through a comprehensive and in-depth analysis of aerogels in the field of CO_2_ capture, this study will provide valuable references for researchers, enabling them to quickly grasp the latest advances in this field and facilitating the promotion of technological innovation and industrial development in this area. This paper systematically summarizes preparation methods such as supercritical drying, ambient pressure drying, and freeze-drying, introduces the classification and design strategies of aerogels, and finally discusses the application status of different aerogel materials in scenarios such as direct air capture, post-combustion flue gas capture, natural gas purification, and carbon storage. The future development trends are prospected, with the aim of providing a reference for the research and development of aerogel applications in the field of carbon dioxide capture.

## 2. Preparation Methods

For aerogels, as nanoporous materials with high porosity, low density and excellent thermal insulation performance, the core of their preparation lies in the drying step after the sol–gel process [13]. The choice of drying technology directly determines the microstructure integrity, mechanical properties and production cost of the final material. At present, the preparation of aerogels mainly relies on three mainstream technologies: supercritical drying, ambient pressure drying and freeze-drying.

### 2.1. Supercritical Drying

**Figure 1:**
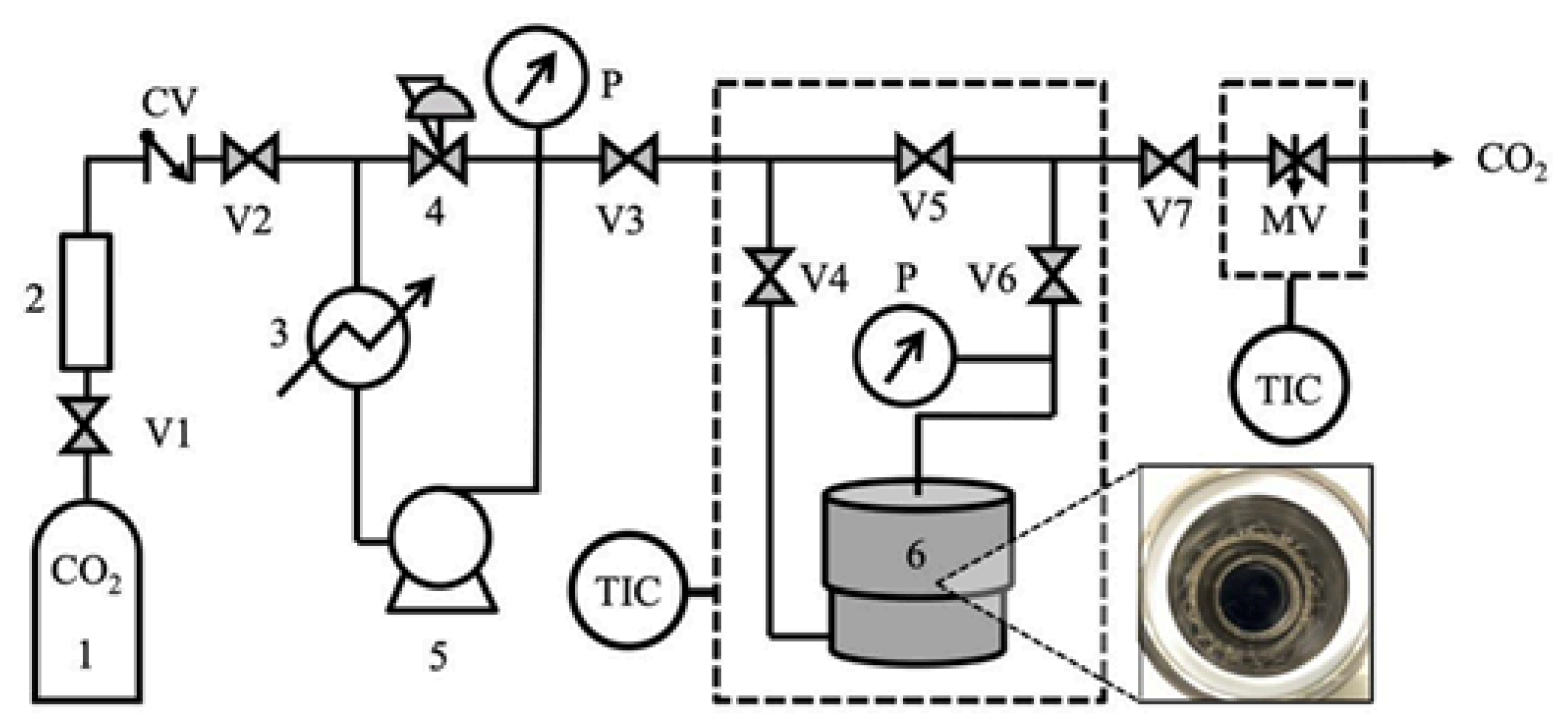
Schematic diagram of supercritical CO_2_ drying equipment: (1) CO_2_ cylinder, (2) water trap, (3) cooler, (4) back pressure regulator, (5) pump, (6) high-pressure chamber, (MV) metering valve, (P) pressure gauge, (TIC) temperature controller, (V1–V7) two-way valves [20]. Copyright 2024, Elsevier.

Supercritical drying is one of the most classic and well-established techniques for preparing high-quality aerogels. The theoretical foundation of this method lies in the unique properties of fluids in the supercritical state (above the critical temperature and pressure), where they possess liquid-like density combined with gas-like low viscosity and negligible surface tension [14,15]. In conventional evaporative drying, the significant capillary pressure generated by solvent evaporation leads to the collapse of the gel network. In contrast, supercritical drying, by circumventing the gas–liquid interface, eliminates the destructive capillary forces, thereby perfectly preserving the three-dimensional nanoporous network structure of the wet gel [16]. In the current field of aerogel fabrication, supercritical carbon dioxide (SCCO_2_) drying has become one of the primary approaches within supercritical drying technologies, owing to its mild conditions and environmentally friendly characteristics [17,18,19]. Kataoka et al. [20] prepared amine-modified silica/carbon (AmSiC) aerogels via supercritical CO_2_ drying, as shown in Figure 1.

This method exploits the mild critical temperature (31.1 °C) and pressure (7.4 MPa) of CO_2_, preserving the porous structure and gel network after drying and thus avoiding pore collapse or network damage caused by surface tension during solvent evaporation, as in thermal drying. Moreover, owing to the high porosity formed by supercritical drying, the aerogels exhibit good thermal insulation performance. Under light irradiation, the generated heat can be effectively localized within the aerogel interior, reducing heat conduction losses and thereby enhancing photothermal desorption efficiency.

He et al. [21] prepared hydrophobic silica aerogels via supercritical drying, with the core objective of eliminating capillary pressure by taking advantage of the absence of a gas–liquid interface in supercritical fluids, thereby fully preserving the nanoporous structure and preventing framework collapse. Pokhrel et al. [22] successfully fabricated cellulose nanofibril (CNF) aerogels by combining emulsion templating with supercritical CO_2_ drying. Compared with conventional freeze-drying, which induces CNF aggregation into lamellar structures, scCO_2_ drying can eliminate gas–liquid interfacial tension and capillary forces, thus preventing structural shrinkage and nanofiber aggregation during the drying process.

The supercritical CO_2_ drying strategy offers significant performance advantages in aerogel preparation and is recognized as a method for producing high-quality, highly stable aerogels [23]. This technique exploits the properties of CO_2_ under supercritical conditions, where it simultaneously exhibits liquid-like density and gas-like low viscosity with zero surface tension, thereby completely eliminating the destructive capillary forces encountered in conventional drying. As a result, it perfectly preserves the original three-dimensional nanonetwork and highly mesoporous/interstitial structure of the gel. This structural retention capability endows the material with exceptionally high porosity and specific surface area—reaching up to 341.0 m^2^/g—which greatly enhances the exposure of active sites, adsorption capacity, and outstanding thermal insulation performance. In terms of application performance, amine modification enables this strategy to exhibit high selectivity in ultra-dilute CO_2_ capture; meanwhile, its high-porosity structure facilitates photothermal localization and reduces heat conduction losses, allowing the photothermal desorption regeneration efficiency to reach 80.8%. However, the high capital cost of high-pressure equipment and the batch-wise production mode severely constrain its industrial scaling and expandability. This inherent limitation has driven the development of ambient-pressure drying technologies, which significantly reduce costs through surface chemistry regulation, thereby serving as a key technological pathway for achieving large-scale application of aerogel-based CO_2_ adsorbents [24,25,26].

### 2.2. Ambient Pressure Drying

**Figure 2:**
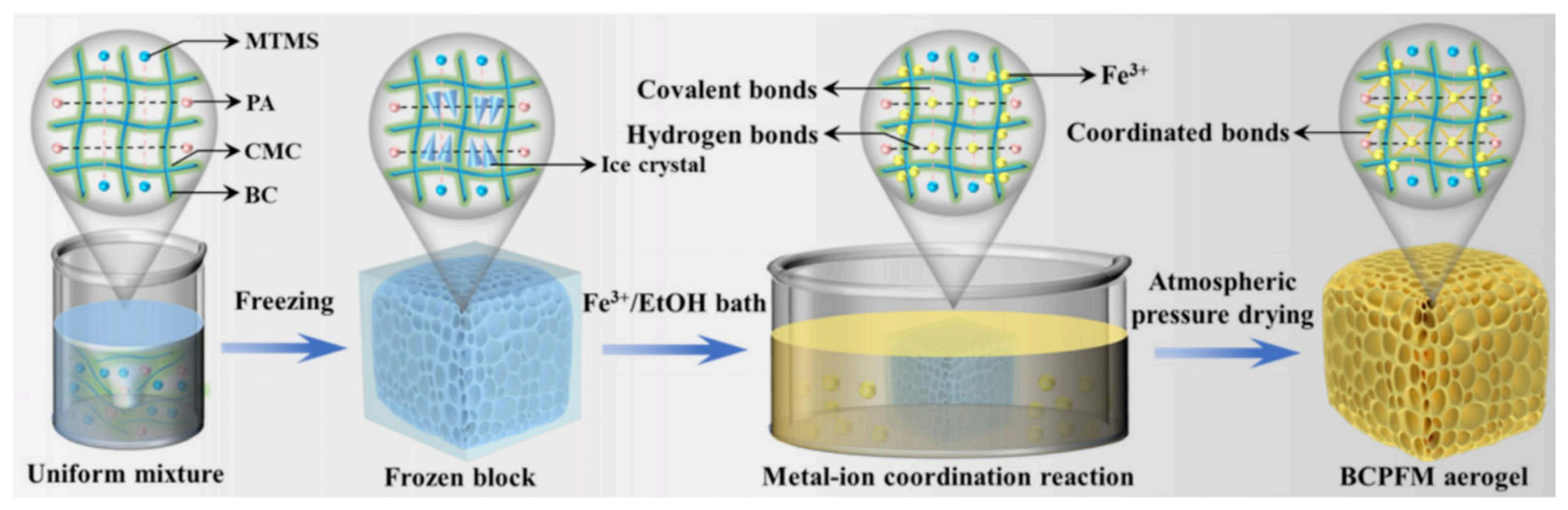
Schematic illustration of the preparation of BCPFM via ambient pressure drying [29]. Copyright 2026, Elsevier.

The core strategy of ambient pressure drying consists of solvent displacement, surface hydrophobic modification, and the utilization of the “spring effect” [27]. Solvent replacement involves substituting water with low-surface-tension solvents such as n-hexane or isopropanol; surface hydrophobic modification involves replacing surface hydroxyl groups with alkyl groups using silane reagents; and the “spring effect” refers to the partial restoration of the gel’s pore structure during drying due to repulsive forces between alkyl groups following hydrophobic modification. The synergistic action of these three steps reduces capillary forces to an acceptable level, thereby lowering production costs at the expense of some loss in specific surface area [28]. To enable the preparation of cellulose aerogels via ambient pressure drying while preserving structural integrity, An et al. [29] innovatively adopted a triple-crosslinking strategy to fabricate flame-retardant thermal insulation cellulose aerogels (BCPFM), as illustrated in Figure 2.

In addition to the above triple cross-linking strategies, some researchers have also optimized the ambient-pressure drying process by introducing various approaches such as ice templating and modifiers, further enhancing the overall performance of aerogels. Zhu et al. [30] prepared aerogels by combining ice templating with ambient pressure drying. Using undissolved cellulose microfibrils (CMFs) as a supporting framework prevented pore structure collapse induced by capillary forces, thereby maintaining structural stability during ambient pressure drying. The dual nanoreinforcement effect of cellulose nanofibers and silica aerogel powder promoted the dispersion of bamboo CMF premixes and the structural robustness of the gel skeleton, allowing the aerogels to be readily constructed via ambient pressure drying. Wang et al. [31] successfully overcame the most intractable issue of structural collapse during ambient pressure drying by introducing the KH-560 modifier to prepare composite aerogels. Despite the use of low-cost ambient pressure drying, the resulting aerogels retained excellent physical properties, even surpassing some literature data obtained via supercritical drying. The product maintained a high porosity of 85.27% and a thermal conductivity as low as approximately 0.03 W·m^−1^·K^−1^.

Ambient-pressure drying exhibits excellent performance in terms of mechanical properties, repeatability, and continuous industrial production [32]. It eliminates the reliance on extreme-temperature-and-pressure equipment [33,34] and offers significant advantages such as low cost, low energy consumption, high safety, and facile shape customization [35]. Although capillary forces during drying tend to cause pore shrinkage and volume loss, which compromise part of the adsorption capacity and mass-transfer rate, precise chemical modification and framework optimization can construct a robust mechanical skeleton, significantly enhancing the compressive strength to 0.20 MPa (at 20% strain) and even reaching 340 kPa. The outstanding structural stability supports production repeatability while maintaining a porosity as high as 85.27%. This strategy, through surface chemistry regulation, represents a key technological pathway for achieving large-scale application of aerogel-based CO_2_ adsorbents.

### 2.3. Freeze Drying

The freeze-drying method involves freezing wet gel to form ice crystals in the solvent, which are then sublimated under vacuum. The voids left after ice crystal sublimation form oriented pore channels [36]. Ice templating is the core mechanism of freeze-drying. By controlling the freezing rate and temperature gradient, the morphology and size of ice crystals can be regulated, thereby customizing the pore structure of the final aerogel [37].

Based on the ice-templating principle described above, Guo et al. [38] employed a one-step freeze-drying-assisted molding technique to prepare multicomponent silica fiber aerogels. During the preparation process, the original pores were occupied by ice crystals, which directly sublimated into water vapor, leaving behind large cavities after sublimation. This approach avoided the damage to the aerogel skeleton caused by the surface tension of liquid water, thereby preserving the nanoporous structure and high porosity of the material. Moreover, increasing the aerogel content facilitated the filling of these cavities. When the aerogel content reached 8%, the fibers were uniformly wrapped by aerogel particles, resulting in a more consistent and refined pore structure that enhanced the overall uniformity of the composite.

**Figure 3:**
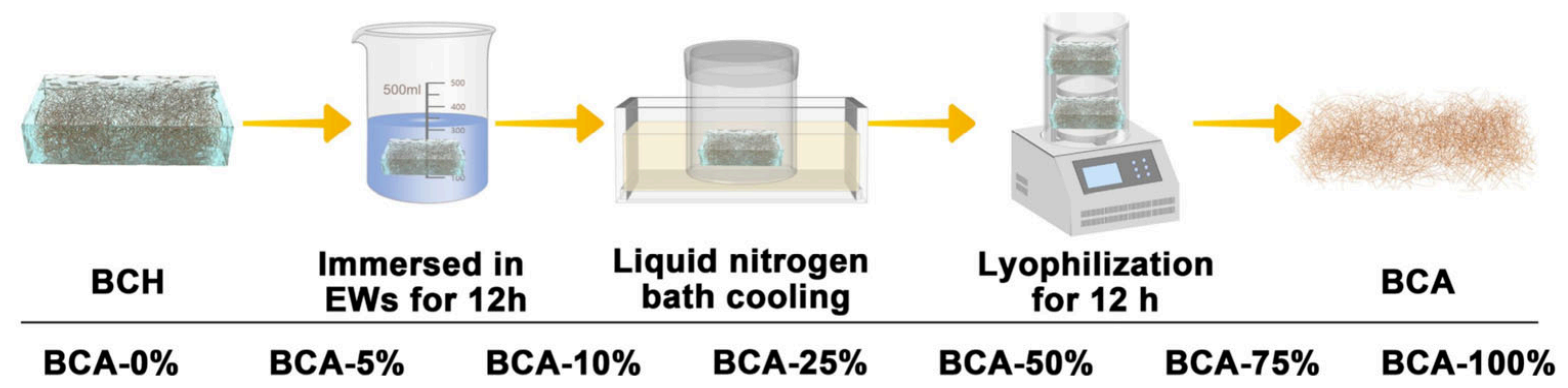
Schematic illustration of the preparation of BCA via the EMFD strategy [39]. Copyright 2025, Elsevier.

To further address the issues of ice crystal damage and structural cracking during conventional freeze-drying, Wei et al. [39] developed a high-performance bacterial cellulose aerogel (BCA) via an ethanol-mediated freeze-drying (EMFD) strategy, as illustrated in Figure 3. Compared with conventional freeze-drying, which suffers from ice-crystal damage, structural collapse, and macroscopic cracking, the mixed solvent of ethanol and water in EMFD lowered the freezing point. During the freeze-drying process, the low-concentration ethanol–water solution formed ice-based solid solutions, which remained stable at the drying chamber temperature and provided a robust physical support, ensuring that the solvent was removed via sublimation rather than evaporation. Meanwhile, capillary stress was alleviated, enabling the fabrication of large-sized, crack-free, and intact aerogel monoliths.

In addition to regulating the solvent system, some researchers have also controlled the ice crystal morphology by increasing the freezing rate. Dong et al. [40] prepared oriented ice-templated aerogel adsorbents using liquid-nitrogen rapid freezing combined with freeze-drying. The ultra-fast freezing rate (>100 °C·min^−1^) suppressed ice crystal growth, forming uniform micron-scale pores (5–20 μm in diameter). The resulting aerogels achieved a CO_2_ adsorption capacity of 2.0 mmol·g^−1^, and even under direct air capture (DAC) conditions (400 ppm CO_2_), an adsorption capacity of 0.8 mmol·g^−1^ was still attainable. The oriented pore channels reduced the half-saturation time of adsorption to within 15 min.

Freeze drying exhibits unique potential for preparing aerogels with controllable pore structures, high porosity, low density, and excellent mechanical properties, particularly supported by improved ethanol-mediated freeze drying (EMFD) and directional ice-templating techniques. This method, through directional ice templating, forms oriented channels (5–20 μm pore size) that significantly reduce gas diffusion resistance, shortening the adsorption half-saturation time to 1.28 min and greatly enhancing mass-transfer kinetics. In terms of adsorption capacity, although conventional freeze drying is susceptible to ice-crystal growth causing structural fragility and loss of nanoporosity, the EMFD strategy employs a low-surface-tension ethanol solution to restrict ice-crystal growth and alleviate capillary stress, achieving a specific surface area as high as 228.7 m^2^/g—far exceeding the 98.1 m^2^/g obtained by the conventional method. Moreover, such aerogels exhibit excellent mechanical properties, such as a compressive strength of 0.5 MPa, and show only a 4% decline in performance after 180 days of operation under humidity-swing conditions, demonstrating good cyclic regeneration stability. Nevertheless, conventional freeze drying still faces challenges such as poor structural integrity and low mechanical strength, requiring careful consideration of solvent selection to avoid structural collapse. Its inherent limitations lie in the long drying cycle (72 h) and relatively low specific surface area, which constrain its preparation efficiency and performance in large-scale continuous production, prompting researchers to continually explore emerging technologies that can strike a new balance among efficiency, cost, and pore structure. A comparative summary of these three techniques is presented in Table 2.

## 3. Classification and Design Strategies of Aerogel Materials

In the context of CO_2_ adsorption research, aerogels can be categorized into four major classes according to their chemical composition: organic, inorganic, carbon-based, and composite aerogels. These materials are typically prepared via sol–gel polymerization, followed by drying processes such as supercritical drying, to preserve their highly porous structures.

### 3.1. Organic Aerogels

**Figure 4:**
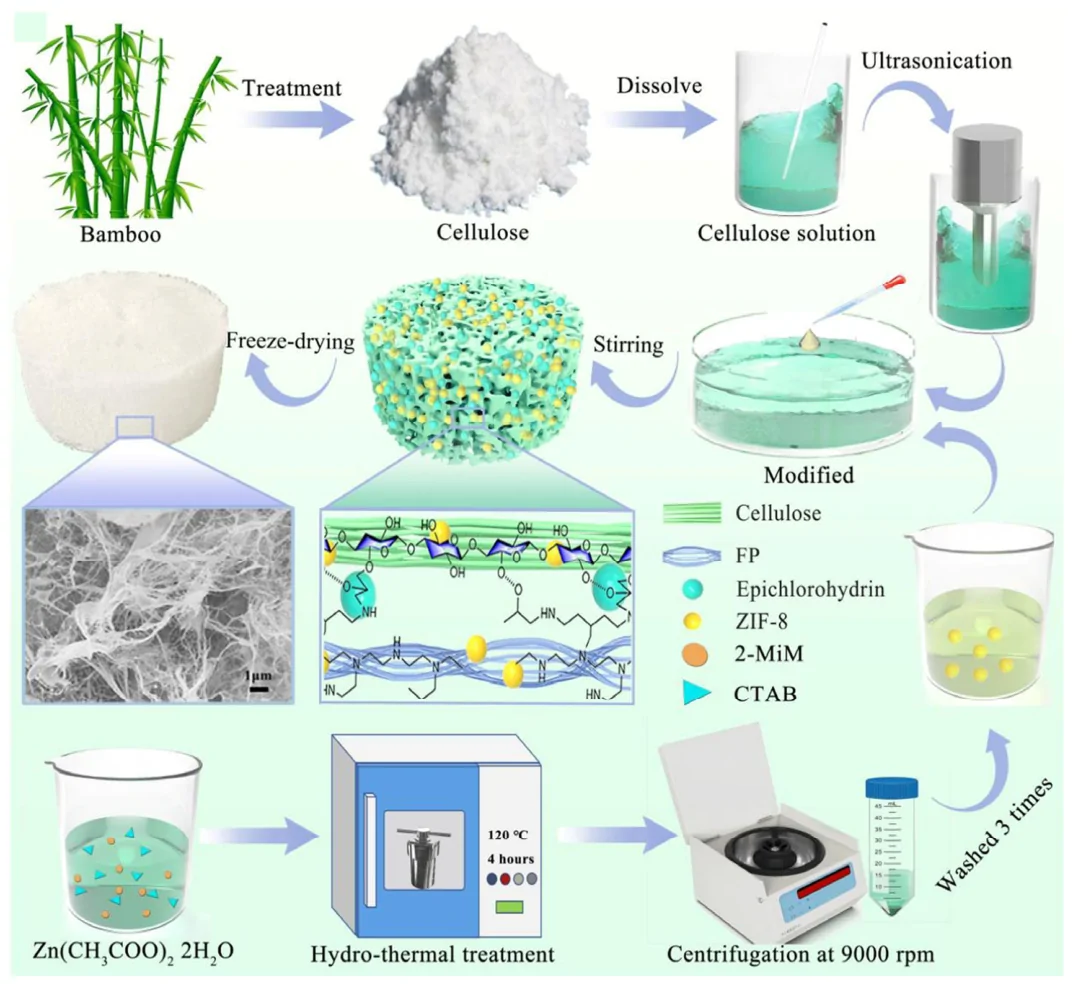
Schematic illustration of the preparation of cellulose aerogel [43]. Copyright 2024, Elsevier.

Organic aerogels are formed by sol–gel polymerization of organic precursors such as resorcinol–formaldehyde (RF), cellulose, chitosan, lignin, and polyurethane [41]. Their skeletons naturally contain abundant oxygen-containing functional groups (hydroxyl, carboxyl, and aldehyde groups), and they exhibit low density, high specific surface area (typically >800 m^2^·g^−1^), and tunable surface chemistry, making them ideal candidates for CO_2_ adsorption applications. However, pristine organic aerogels exhibit very low physisorption capacities (<0.5 mmol·g^−1^); after amine functionalization, the adsorption capacity can be enhanced by 2–5 times [42]. Chen et al. [43] fabricated a cellulose-based aerogel (ZFPCa) for synergistic CO_2_ adsorption by using bamboo-derived cellulose as the substrate and incorporating functionalized PEI and zeolitic imidazolate framework-8 (ZIF-8) via a one-step freeze-drying method, as shown in Figure 4. In this study, ZIF-8 served as a supporting layer to mitigate the aggregation effect of PEI molecules, thereby providing a stable porous structure and enhancing adsorption capacity. The maximum CO_2_ adsorption capacity reached 9.26 mmol·g^−1^, and after 10 adsorption–desorption cycles, the capacity remained at 7.13 mmol·g^−1^.

In addition to introducing fillers as a synergistic reinforcing phase, some researchers have also explored different pathways for performance enhancement by starting from the pore structure and preparation process optimization of the organic aerogels themselves. Bakshi et al. [44] prepared porous organic aerogels via freeze-drying. The excellent porosity of these aerogels provided abundant physical space and transport channels for CO_2_ molecules, constructing a porous framework with efficient CO_2_ capture capability. The adsorption capacity ranged from 0.2440 to 2.4764 mmol·g^−1^, with equilibrium times ranging from 6.10 to 11.21 min. Hsan et al. [45] prepared organic aerogels via a stepwise freeze-drying method, utilizing their abundant mesoporous structures for physical filling, and synergistically exploiting the strong acid–base chemical interaction between basic amine groups and acidic CO_2_ to form carbamates, achieving efficient capture with a CO_2_ adsorption capacity of 5 mmol·g^−1^.

Organic aerogels exhibit significant advantages in CO_2_ capture, with their core competitiveness predominantly attributable to high porosity and specific surface area, which provide abundant active sites for gas adsorption. Furthermore, their bio-based precursors endow the materials with excellent renewability, biodegradability, and considerable cost-reduction potential. Through amination modification or MOFs, the surface chemical properties and three-dimensional network structure can be precisely regulated, thereby achieving a synergistic enhancement of physical and chemical adsorption and significantly improving the thermal stability and mechanical strength of the material. However, organic aerogels are limited by their thermal stability (decomposition temperature is usually less than 300 °C), and they may face the risk of skeleton degradation in high-temperature flue gas capture scenarios. This has prompted a shift towards researching inorganic aerogel systems with better thermal stability.

### 3.2. Inorganic Aerogels

Inorganic aerogels are prepared by the sol–gel method using metal oxides such as SiO_2_ as the framework and metal alkoxides or inorganic salts [46]. The Si–O–Si inorganic skeleton endows these materials with excellent thermal stability (>500 °C) and mechanical robustness. The specific surface area of pure SiO_2_ aerogel can reach 800 to 1200 m^2^·g^−1^, but its surface is mainly composed of silanol groups, which only weakly adsorb CO_2_. Under conditions of 25 °C and 1 bar, the adsorption capacity is usually less than 0.5 mmol·g^−1^ [47]. Therefore, the improvement in the CO_2_ adsorption performance of inorganic aerogels depends on amino functionalization. The core challenge lies in the fine balance between the density of amino functionalization and the accessibility of pore channels. Although an excessively high amine loading increases the chemical adsorption sites, it will clog the mesoporous channels, resulting in a decrease in the effective specific surface area and an increase in diffusion resistance.

**Figure 5:**
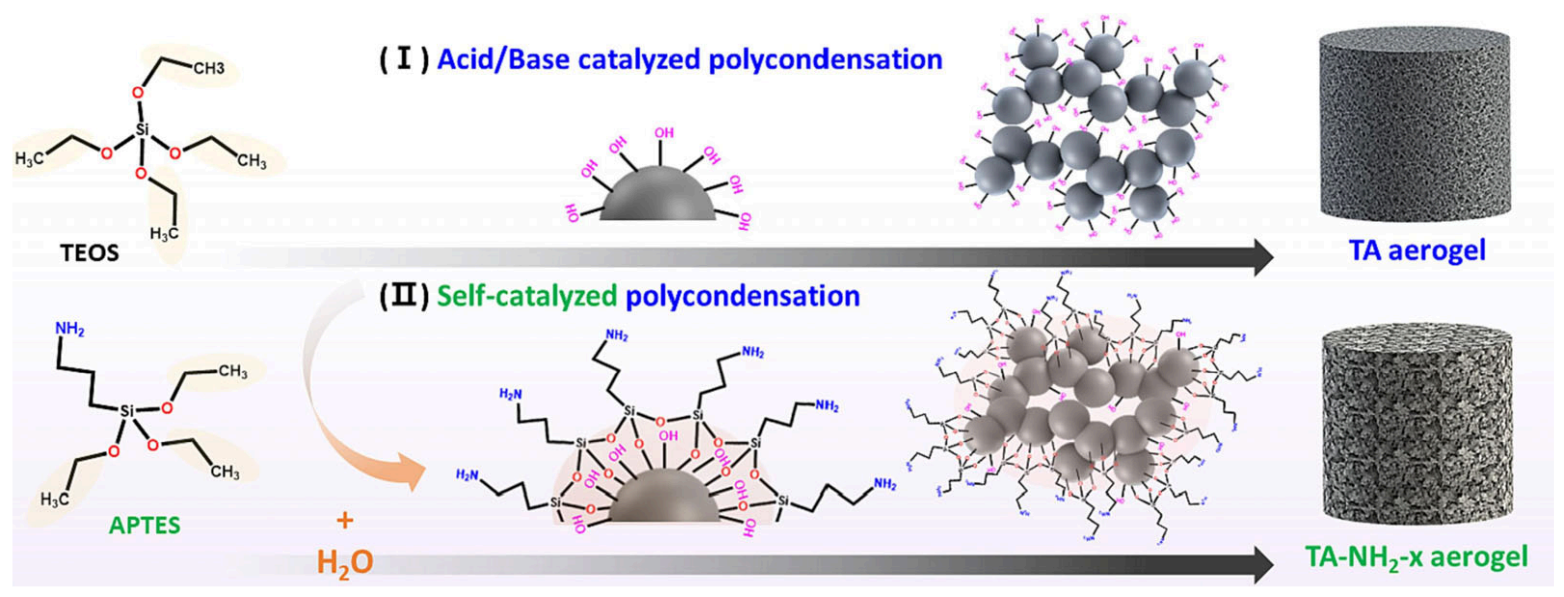
Schematic illustration of the reaction mechanism of the aerogel [45]. Copyright 2024, Elsevier.

Choi et al. [48] reported rigid aromatic amine-grafted SiO_2_ aerogels, in which the aromatic-ring-containing rigid amine structures resist pore collapse. Even at an amine loading as high as 40 wt%, the aerogels retained a high specific surface area (>600 m^2^·g^−1^). The CO_2_/N_2_ selectivity, calculated using the IAST method, exceeded 100, and the material maintained over 85% of its adsorption capacity under humid flue gas conditions, as illustrated in Figure 5. Wang et al. [49] optimized the adsorption conditions for amine-modified SiO_2_ aerogels, investigating the interactive effects of amine loading (20–50 wt%), adsorption temperature (25–75 °C), and CO_2_ partial pressure (0.1–1 bar). The optimal conditions were identified as an amine loading of 35 wt% and an adsorption temperature of 50 °C, yielding a CO_2_ adsorption capacity of 2.0 mmol·g^−1^, with an isosteric heat of adsorption (Qst) ranging from 42 to 68 kJ·mol^−1^. Joo et al. [50] employed liquid-nitrogen freeze-drying and introduced Ti(OH)_4_ as a functional additive to construct silica aerogels, achieving a maximum adsorption capacity of 4 mmol·g^−1^. This aerogel forms carbamate or bicarbonate through the chemical reaction of amino groups with CO_2_, and utilizes the abundant hydroxyl groups provided by Ti(OH)_4_ to strengthen the hydrogen bond network, thereby synergistically promoting the capture and stabilization of CO_2_.

Inorganic aerogels such as SiO_2_ aerogels [51], as CO_2_ adsorption materials, have the advantages of high specific surface area and unique nano-three-dimensional network structure. Moreover, the Si-O-Si inorganic skeleton endows them with excellent thermal stability. After amino functionalization modification, the transformation from weak physical adsorption to enhanced chemical adsorption can be achieved, significantly enhancing the adsorption capacity and CO_2_/N_2_ selectivity. However, the adsorption capacity of pure materials is weak, and during the modification process, there is a contradiction between the amine loading density and the accessibility of the pore channels. Excessive loading can easily lead to pore blockage and increased diffusion resistance. In addition, the preparation process usually relies on expensive supercritical drying technology, which is complex and costly, thus limiting its large-scale industrial application.

### 3.3. Carbon-Based Aerogels

Carbon-based aerogels include carbon aerogels, graphene aerogels, carbon nanotube (CNT) aerogels, and biomass carbon aerogels, etc., and are usually prepared from organic aerogel precursors through high-temperature carbonization or hydrothermal carbonization [52,53,54]. The carbon skeleton endows aerogels with electrical conductivity, chemical stability and a rich microporous structure [55], among which the volume of micropores is a key parameter determining the physical adsorption capacity of CO_2_ [56]. Unlike organic and inorganic aerogels, the CO_2_ adsorption of carbon-based aerogels can be achieved through the synergistic effect of microporous physical adsorption and N-doped chemical adsorption, and the conductivity of the carbon skeleton provides a unique advantage for Joule thermal regeneration [57].

Based on the aforementioned multiple adsorption advantages of carbon-based aerogels, Li et al. [58] adopted a typical preparation method for carbon-based aerogels by pyrolyzing phosphoric acid-impregnated cotton at high temperature under a nitrogen flow to form a twisted carbon fiber aerogel with structural integrity. This aerogel possesses a highly developed microporous structure, and the abundant micropore volume provides a large number of adsorption sites for CO_2_ molecules, while the surface C–O and O–H functional groups also participate in the CO_2_ adsorption process.

In addition to direct carbonization of biomass, constructing ordered structures in advance via directional freeze-drying followed by carbonization can further enhance adsorption performance from the perspective of mass transfer kinetics. Bao et al. [59] prepared an elastic carbon nanofiber aerogel by directional freeze-drying technology, with a CO_2_ capture capacity as high as 4.88 mmol/g. The ordered vertical honeycomb channels facilitate CO_2_ diffusion and transport within the material, enhancing adsorption kinetics. Capture is driven by van der Waals forces between the adsorbent surface and CO_2_ molecules, while loaded amine groups react with CO_2_ to form carbamates, thereby immobilizing CO_2_.

**Figure 6:**
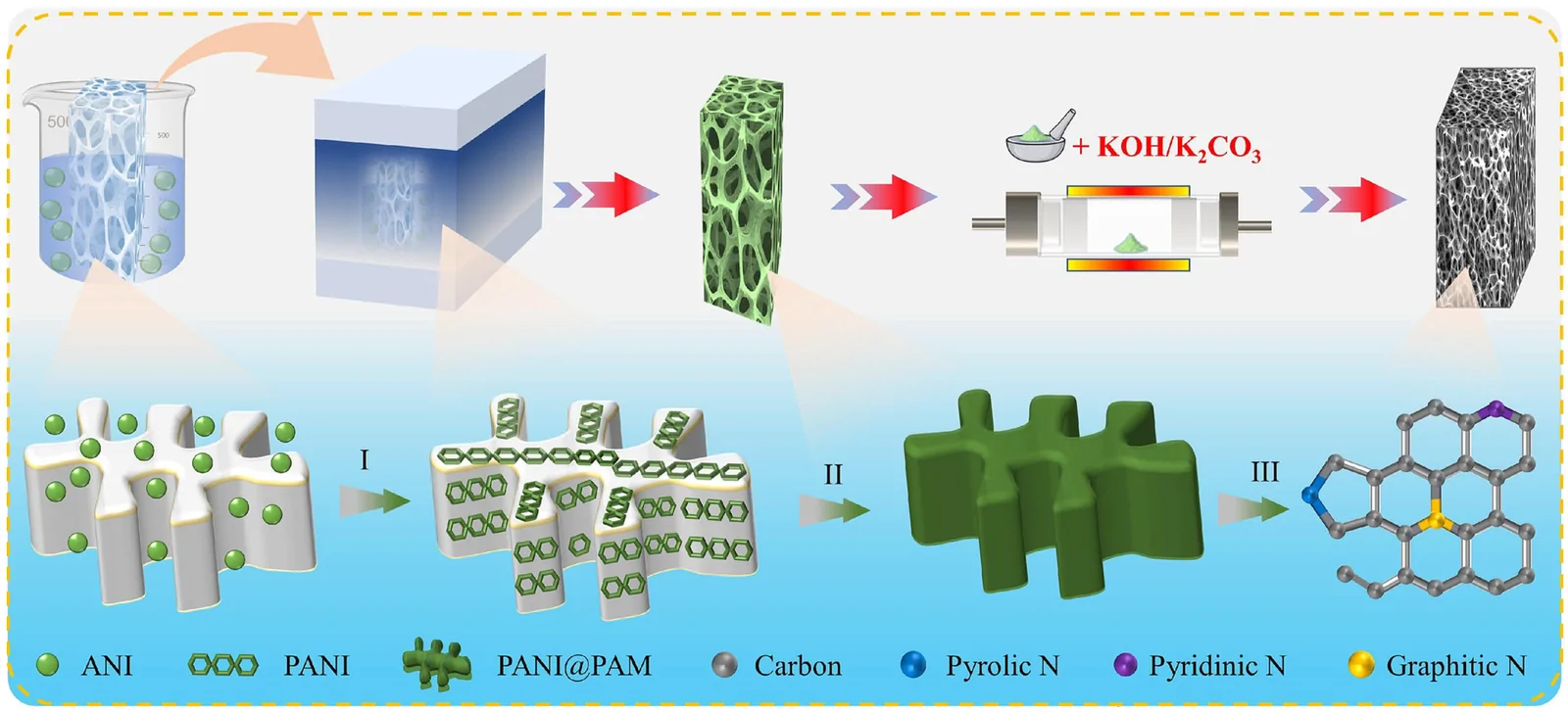
Synthesis and formation mechanism of the aerogel [60]. Copyright 2025, Elsevier.

In order to simultaneously achieve the synergistic optimization of rapid diffusion and strong capture, Zhang et al. [60] constructed three-dimensional interconnected porous carbon aerogels through low-temperature polymerization followed by high-temperature calcination and activation, as depicted in Figure 6. The interconnected macro-/mesoporous channels promote rapid gas diffusion, while micropores provide abundant adsorption sites for efficient capture through enhanced van der Waals forces. In addition, strong Lewis acid–base interactions and electrostatic attractions exist between basic sites in the aerogel and acidic CO_2_ molecules, and surface oxygen-containing groups with high electron density donate electron density to the electron-deficient carbon atoms of CO_2_, further strengthening the interactions.

The core advantages of carbon-based aerogels lie in the synergistic effect of microporous physisorption and N-doping chemisorption, as well as the electrically conductive regeneration capability imparted by the carbon skeleton. However, under high-pressure conditions, the volume increase after micropore filling saturation is limited, and the pore structure shrinkage during the carbonization process needs to be precisely controlled, usually ranging from 20% to 40%. While carbon-based aerogels excel in microporous physisorption and electro-regeneration, single-component systems struggle to simultaneously meet the multi-objective requirements of high selectivity, high capacity, and low regeneration energy consumption. Composite aerogels are expected to break through the performance ceiling of a single material by introducing materials such as MOF, Ti_3_C_2_T_x_, and PEI.

### 3.4. Composite Aerogels

#### 3.4.1. MOF Composites

Metal–organic frameworks (MOFs) are crystalline porous materials formed by self-assembly of metal ions/clusters and organic ligands through coordination bonds [61]. Among them, metal nodes act as connection points, and organic ligands connect them like crossbeams, forming a highly ordered three-dimensional network structure, which contains a large number of regularly arranged channels inside [62]. MOFs feature a high specific surface area and adjustable pore size and chemical functions, and have diverse structures, covering a wide range of application requirements [63,64,65].

**Figure 7:**
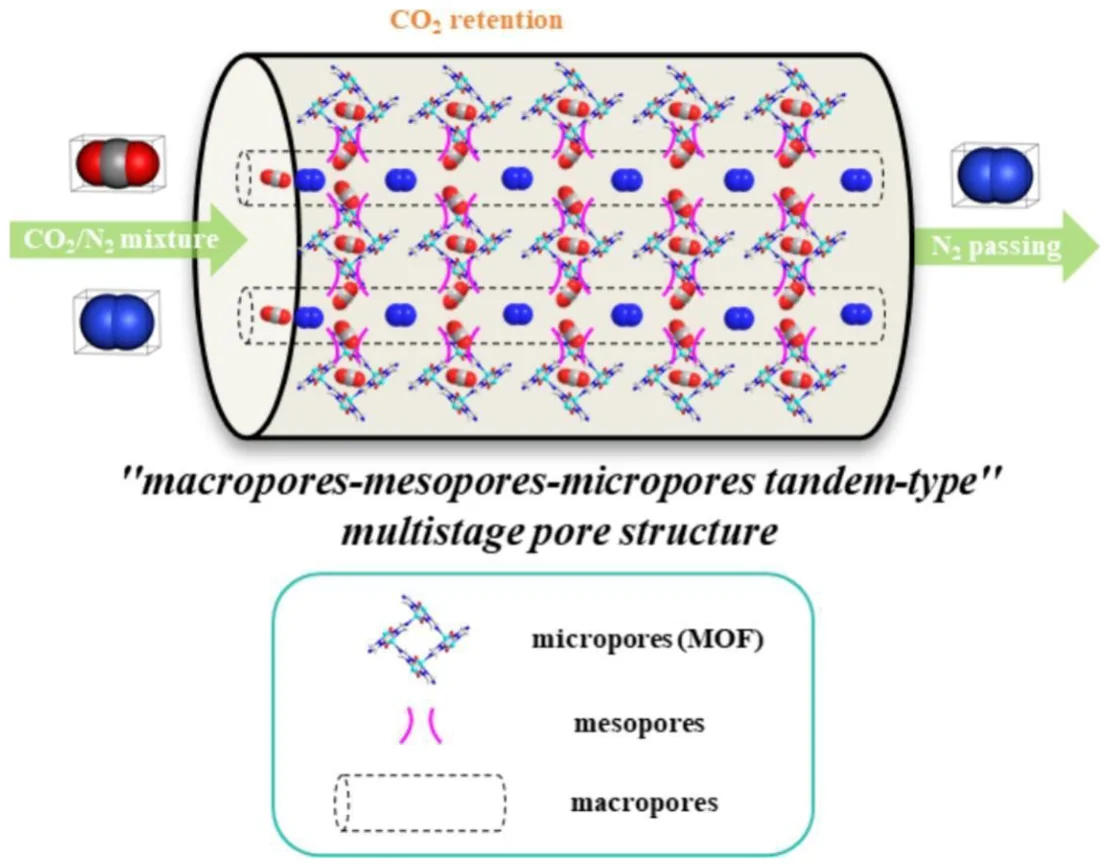
Schematic of the aerogel structure and CO_2_ separation [66]. Copyright 2025, Elsevier.

Che et al. [66] prepared a composite aerogel by freeze-drying using two isomeric zinc-based metal–organic frameworks for CO_2_ capture and separation, as shown in Figure 7. The aerogels constructed a hierarchical “macro–meso–micro” pore architecture that enabled efficient adsorption. This three-dimensional network not only enhanced mass transfer but also distributed stress through cross-linked skeletons, improving mechanical strength. Yao et al. [67] successfully prepared a multifunctional CO_2_-responsive magnetic aerogel (Ag/Fe-NC@DCG) by freeze-drying. MOF-derived Ag/Fe nanocarbons were embedded into cellulose nanocrystal (CNC)-reinforced gelatin, and the aerogel was functionalized with N, N-diethyl-3-aminopropyltrimethoxysilane (DATMS). In this work, the MOF served as a precursor, converting to Ag/Fe nanocarbons upon carbonization, thereby imparting photo-Fenton catalytic activity and magnetism to the composite aerogel, while avoiding complex sedimentation or filtration steps and enhancing recyclability and reusability. Zhang et al. [68] prepared lignin-regulated MOF@CNFs/DESL30G aerogels. Using lignocellulosic aerogel as a substrate, MOFs were uniformly and robustly integrated via in situ growth. Although MOF powders suffer from poor mechanical strength, a lack of self-support, and difficulties in processing and recovery, immobilizing and dispersing them on suitable substrates can enhance composite properties, overcoming these limitations and promoting practical application in carbon capture systems.

MOF composite aerogels inherit the intrinsically tunable microporosity and ultra-high specific surface area of MOFs, exhibiting excellent adsorption performance, including high CO_2_ selectivity. In addition, these aerogels offer tunable adsorption capacity, enhanced mechanical strength and structural stability, and good reusability. However, the composite process may lead to reductions in specific surface area and adsorption capacity, and catalytic active sites may partially deactivate after repeated use. The relatively complex preparation process and the risk of adsorption saturation upon over-filling also require further investigation and resolution.

#### 3.4.2. Ti_3_C_2_T_x_ Composites

Ti_3_C_2_T_x_ is the most extensively studied two-dimensional layered nanomaterial within the MXene family, belonging to the class of transition metal carbides [69]. Its general chemical formula is M_n+1_X_n_T_x_, where M represents an early transition metal (Ti in this case), X is carbon, and T_x_ denotes surface-terminating functional groups, such as –O, –OH, and –F, which originate from the etching process [70]. Ti_3_C_2_T_x_ possesses high electrical conductivity, a high specific surface area, abundant surface functional groups, and favorable electrochemical activity, rendering it widely applicable in the field of composite aerogels [71].

**Figure 8:**
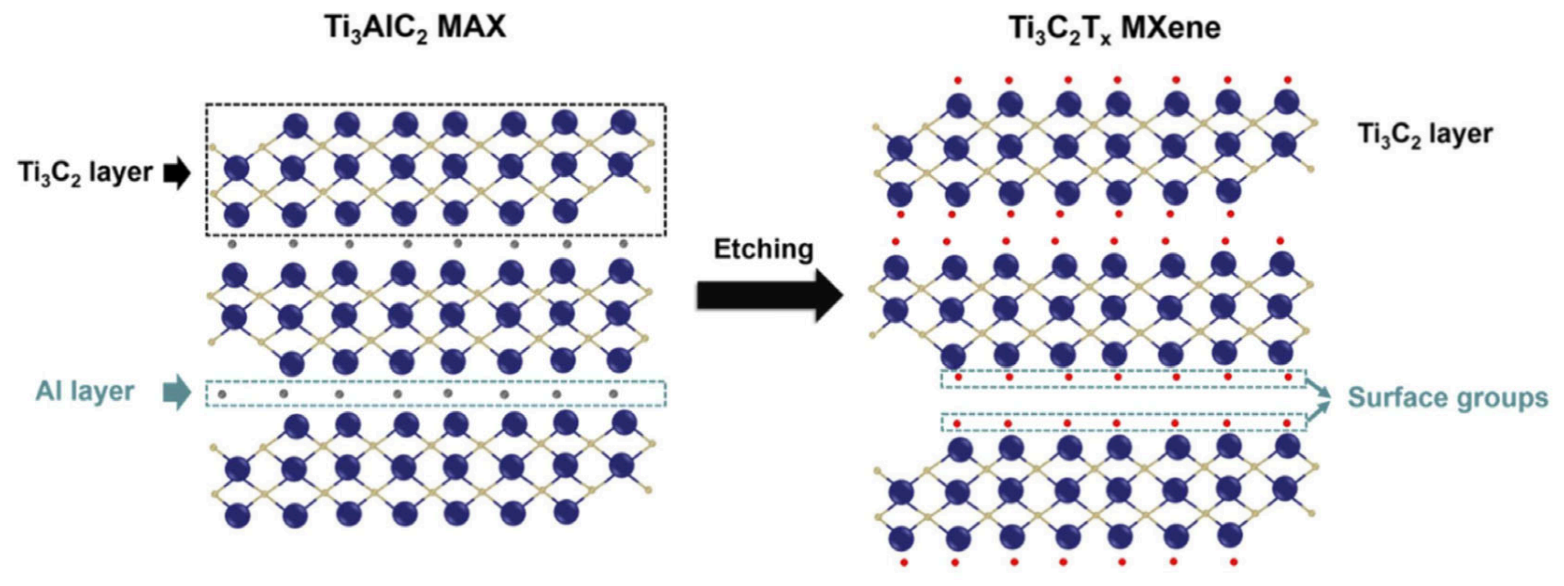
Schematic illustration of the synthesis of Ti_3_C_2_T_x_ MXene [74]. Copyright 2026, Elsevier.

Chong et al. [72] prepared a bifunctional Ti_3_C_2_T_x_/graphene aerogel (GA)/TiO_2_ (TGT) ternary composite aerogel and subjected it to amino modification. This aerogel was employed in a photo-enzyme coupled system (PECS) for the reduction of CO_2_ to formic acid. The large two-dimensional surface and good CO_2_ affinity of Ti_3_C_2_T_x_ enable its simultaneous participation in both photocatalytic and enzymatic reactions, thereby achieving effective CO_2_ adsorption and conversion. Li et al. [73] fabricated CsPbBr_3_/MXene composite aerogels by immobilizing pre-synthesized CsPbBr_3_ nanocrystals (NCs) onto MXene hydrogels, followed by freeze-drying. Ti_3_C_2_T_x_ not only provides structural support but also synergistically enhances light harvesting, charge separation and transport, and CO_2_ activation through its superior electrical conductivity, adsorption capacity, and abundant catalytic sites, ultimately significantly improving the efficiency and stability of photocatalytic CO_2_ reduction. Meng et al. [74] prepared anisotropic polyimide (PI)/Ti_3_C_2_T_x_ MXene composite aerogels via unidirectional freeze-drying. Within the polyimide composite aerogel, Ti_3_C_2_T_x_ primarily reinforces the mechanical properties, structural integrity, hydrophobicity, thermal insulation, and flame retardancy through strong hydrogen-bonding interactions, while also modulating the pore structure. Figure 8 presents the schematic of its preparation.

Ti_3_C_2_T_x_ composite aerogels effectively promote CO_2_ reduction to formic acid in photo-enzyme coupled systems, demonstrating excellent photocatalytic activity and enzyme activity recovery. These outstanding performances are attributed to the unique high specific surface area, porous structure, superior electronic conductivity, good CO_2_ affinity, and strong hydrogen-bonding interactions with the matrix material. However, Ti_3_C_2_T_x_ composite aerogels may suffer from interfacial defects, and an excessive amount of Ti_3_C_2_T_x_ can compromise the specific surface area and induce framework shrinkage. In addition, the hydrophilicity of MXene makes its uniform dispersion in certain matrices (such as PI) still challenging, and the strong reducing electrons may lead to the inactivation of enzyme active centers. The loss of particles during the recycling process is also a problem.

#### 3.4.3. Polyethyleneimine Composites

Polyethyleneimine (PEI) is a cationic polymer rich in amino groups, and its molecular skeleton is composed of repeated ethyleneimine units [75]. It is precisely this rich array of active amino functional groups that endows PEI with excellent chemical reactivity, chelating ability and positive charge characteristics [76]. Owing to its high density of primary and secondary amines, PEI simultaneously serves as a chemical crosslinker, a flexible toughening component, and a provider of functional active sites in composite aerogels [77].

**Figure 9:**
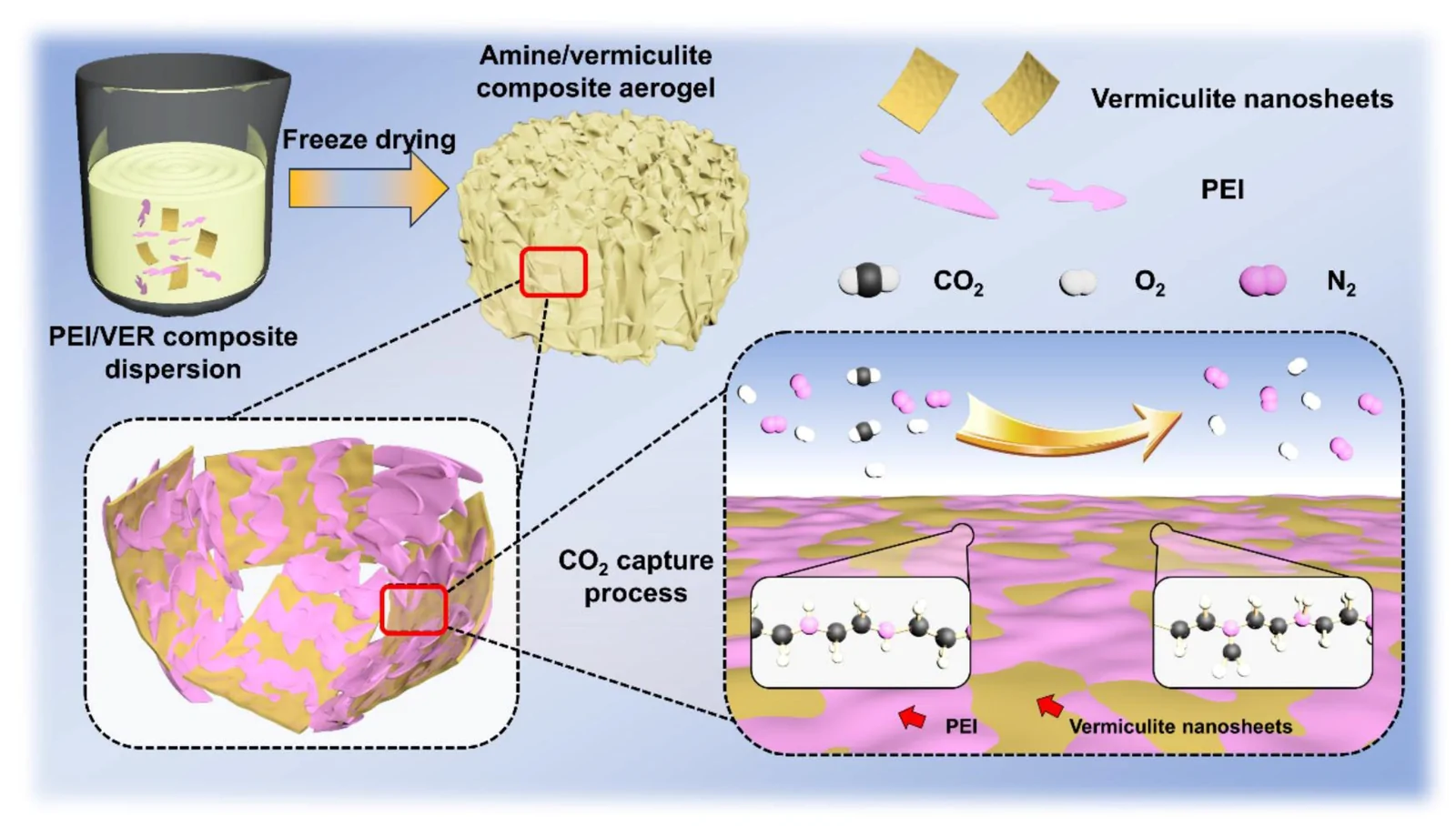
Schematic illustration of aerogel preparation and CO_2_ capture [78]. Copyright 2024, Elsevier.

Wang et al. [78] prepared amine/vermiculite composite aerogels via freeze-drying for CO_2_ capture from ambient air, as shown in Figure 9. PEI acts as a crosslinking agent to promote gelation, while concurrently providing CO_2_ adsorption sites, enhancing amine stability and reducing loss, regulating the aerogel structure, and increasing CO_2_ capture capacity. The amines form chemical bonds by donating electron pairs to CO_2_, thereby enabling selective capture of low-concentration CO_2_ from ambient air, achieving a maximum CO_2_ capture capacity of 0.72 mmol·g^−1^ and an amine efficiency of 0.29. Sami et al. [79] investigated an amine-loaded fly ash incorporated into a polyacrylamide-based stable aerogel. PEI, with its high nitrogen content and low volatility, significantly enhanced the CO_2_ adsorption capacity of the polyacrylamide-based aerogel upon impregnation (4.83 mmol·g^−1^). The PEI-impregnated aerogel surface was covered with amines capable of forming chemical bonds with CO_2_ molecules to generate carbamates. Additionally, the introduction of fly ash increased PEI loading and improved the thermal stability of the aerogel. Ai et al. [80] prepared PEI-functionalized reduced graphene oxide (rGO) aerogels. PEI was utilized as the capture agent due to its high amine density, thermal stability, and commercial availability; its amine groups chemically react with CO_2_, thereby realizing capture.

PEI composite aerogels exhibit high efficiency for CO_2_ capture, favorable cyclic stability, and good regeneration performance. With suitable substrates, they also offer low cost and environmental friendliness. However, the optimization of PEI loading, the selection of molecular weight, and the balance between adsorption capacity, amine efficiency, and pressure drop remain key issues to be addressed in future research.

Through structural design and modification, composite aerogels have achieved progress in CO_2_ capture efficiency, selectivity, and mechanical strength, realizing a “1 + 1 > 2” synergistic effect and demonstrating significant potential for industrial applications [81,82,83]. A comparison of specific composite materials is summarized in Table 3.

## 4. Applications in CO_2_ Adsorption

### 4.1. Direct Air Capture

Direct air capture (DAC) represents the most challenging scenario for CO_2_ capture, as the atmospheric CO_2_ concentration is only approximately 420 ppm (corresponding to a partial pressure of about 0.04 kPa), which is considerably lower than that of any industrial emission source [84,85,86]. Under such low partial pressures, the driving force for physisorption is weak (Qst typically < 25 kJ·mol^−1^), necessitating a strong chemisorption driving force (Qst > 50 kJ·mol^−1^) to achieve thermodynamically effective capture. Simultaneously, the adsorbent must exhibit high CO_2_ selectivity in the presence of abundant N_2_ (78%) and O_2_ (21%), and maintain long-term stability under temperature–vacuum swing adsorption (TVSA) cycles [87].

**Figure 10:**
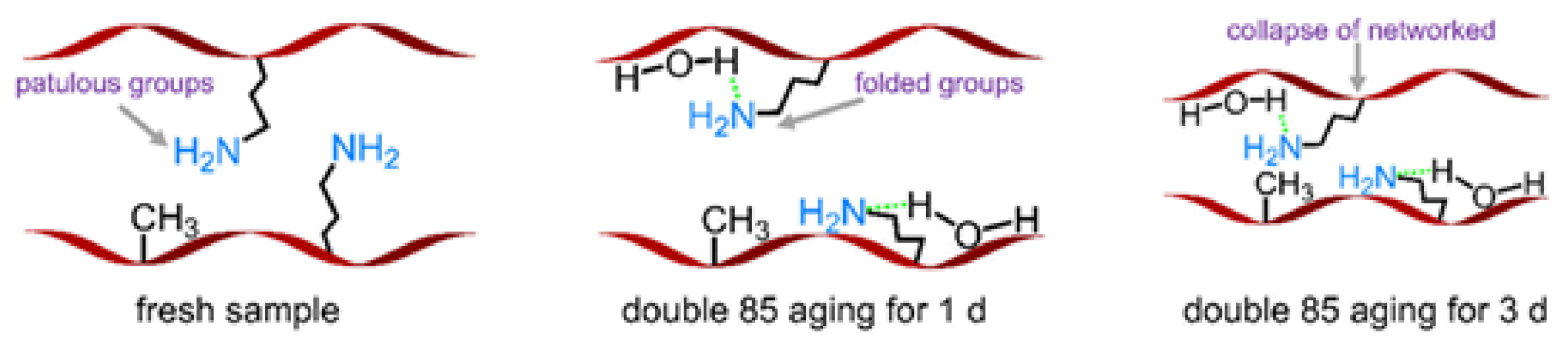
Schematic illustration of the aerogel structure [88]. Copyright 2025, Elsevier.

In response to the dual challenges of low CO_2_ concentration and high humidity in the DAC process, starting from hydrophobic modification and adsorption site regulation, different functionalized aerogel design strategies have been explored. To address the above pain points of low-concentration adsorption difficulty, high energy consumption, and poor stability, Kong et al. [88] adopted supercritical drying to develop a hydrophobic amine-functionalized silica aerogel, as shown in Figure 10. The specific surface area, for example, of SA-AM-M reached 306 m^2^/g, which promoted full exposure of surface amine functional groups and enhanced the collision frequency between CO_2_ and amine groups under low partial pressure. In addition, the introduction of hydrophobic –CH_3_ groups, with a water contact angle of 133°, not only protected the nano-skeleton from capillary-force-induced collapse, but also reduced competitive adsorption of water molecules, thereby ensuring the stability of amine active sites in humid air. This aerogel achieved an adsorption capacity of 0.97–1.14 mmol/g at 25 °C under DAC conditions, and its well-developed nanoporous network significantly improved adsorption kinetics. The regeneration temperature was only 65 °C, which can substantially reduce industrial operating costs. Meanwhile, with its strongly hydrophobic surface (contact angle 133°), the material exhibited excellent durability in humid air, maintaining robust performance after 18 months of simulated operation, thus providing an ideal material solution for low-cost, long-life industrial air carbon capture.

Beyond the supercritical drying route, Ziemiański et al. [89] attempted to prepare hydrophobic amine-functionalized aerogels via the more economical ambient-pressure drying process and achieved further improvements in water resistance and selectivity. This material exhibited outstanding performance in simulated real-air (400 ppm CO_2_) capture experiments, not only possessing a superhydrophobic contact angle as high as 161° and reducing the water uptake at 95% relative humidity to below 6 wt%, but also showing a CO_2_/H_2_O selectivity 10–25% higher than that of a commercial benchmark resin at 80% humidity. Its excellent dimensional stability and constant adsorption kinetics even after water saturation, combined with the low-cost ambient-pressure drying preparation process, provide a promising material solution for achieving efficient and low-energy-consumption large-scale direct air carbon capture.

In addition to resisting moisture interference through hydrophobic modification, there is also the approach of converting humidity itself into energy to drive the CO_2_ cycle. Wang et al. [90] prepared three functionalized cellulose aerogels QCA, GQCA, MSQCA and a CER-MSQCA composite aerogel using various drying techniques. By employing a humidity-swing mechanism, they achieved efficient CO_2_ capture from the atmosphere through reversible hydration reactions involving adsorption under dry conditions and desorption under high-humidity conditions. Among these, the MSQCA aerogel, benefiting from its open 3D hierarchical pore structure and additional adsorption sites introduced via maleic acid crosslinking, attained an adsorption capacity of 2.37 mmol·g^−1^. The further optimized CER-MSQCA composite aerogel achieved an ultra-high capture capacity of 3.71 mmol·g^−1^ and fast kinetics (half-life of only 1–2 h), while exhibiting excellent mechanical robustness over 30 dry–wet cycles.

In response to the extreme challenges of ultra-low CO_2_ concentrations (approximately 420 ppm) and environmental moisture competition in DAC, the structural design strategies for aerogels focus on enhancing the chemical adsorption driving force and interfacial hydrophobic regulation. Through the synergy of introducing high-density amine functional groups and high specific surface area (e.g., 306 m^2^/g), the collision frequency of dilute gases is effectively increased, enabling the modified silica aerogel to still achieve a capacity of 0.97–1.14 mmol/g at 400 ppm. To address the risks of competitive adsorption and framework collapse caused by the partial pressure of water being much higher than that of CO_2_ under high humidity conditions, the use of long-chain alkyl or silane hybrid grafting can achieve a superhydrophobic contact angle as high as 161°, reducing water uptake to below 6 wt%, and through interfacial geometric shielding effects, the CO_2_/H_2_O selectivity is improved by 10–25% compared with commercial resins. In addition, employing the moisture swing mechanism to drive the reversible conversion of hydration reactions can turn a hazard into a benefit, achieving an ultra-high capture capacity of 3.71 mmol/g, while ensuring mechanical robustness over 30 cycles and significantly reducing the operational energy consumption of DAC through an ultra-low regeneration temperature of 65 °C. DAC represents the most extreme scenario with the lowest CO_2_ concentration and the greatest thermodynamic challenges [91], and when the CO_2_ concentration increases to the flue gas level of 4–15%, the challenges for aerogel adsorbents shift from “insufficient thermodynamic driving force” to “impurity interference and selective competition”.

### 4.2. Post-Combustion Flue Gas Capture

Post-combustion flue gas CO_2_ capture is currently the largest carbon capture application scenario. The flue gas from coal-fired and gas-fired power plants contains 4% to 15% CO_2_ (with a partial pressure of 4 to 15 kPa) and a temperature of 40 to 80 °C [92]. The complexity of flue gas lies in the simultaneous presence of SO_2_, NO and water vapor. Therefore, aerogel adsorbents under flue gas conditions are confronted with three major challenges: CO_2_ selectivity, resistance to impurity interference, and regeneration energy consumption [93].

**Figure 11:**
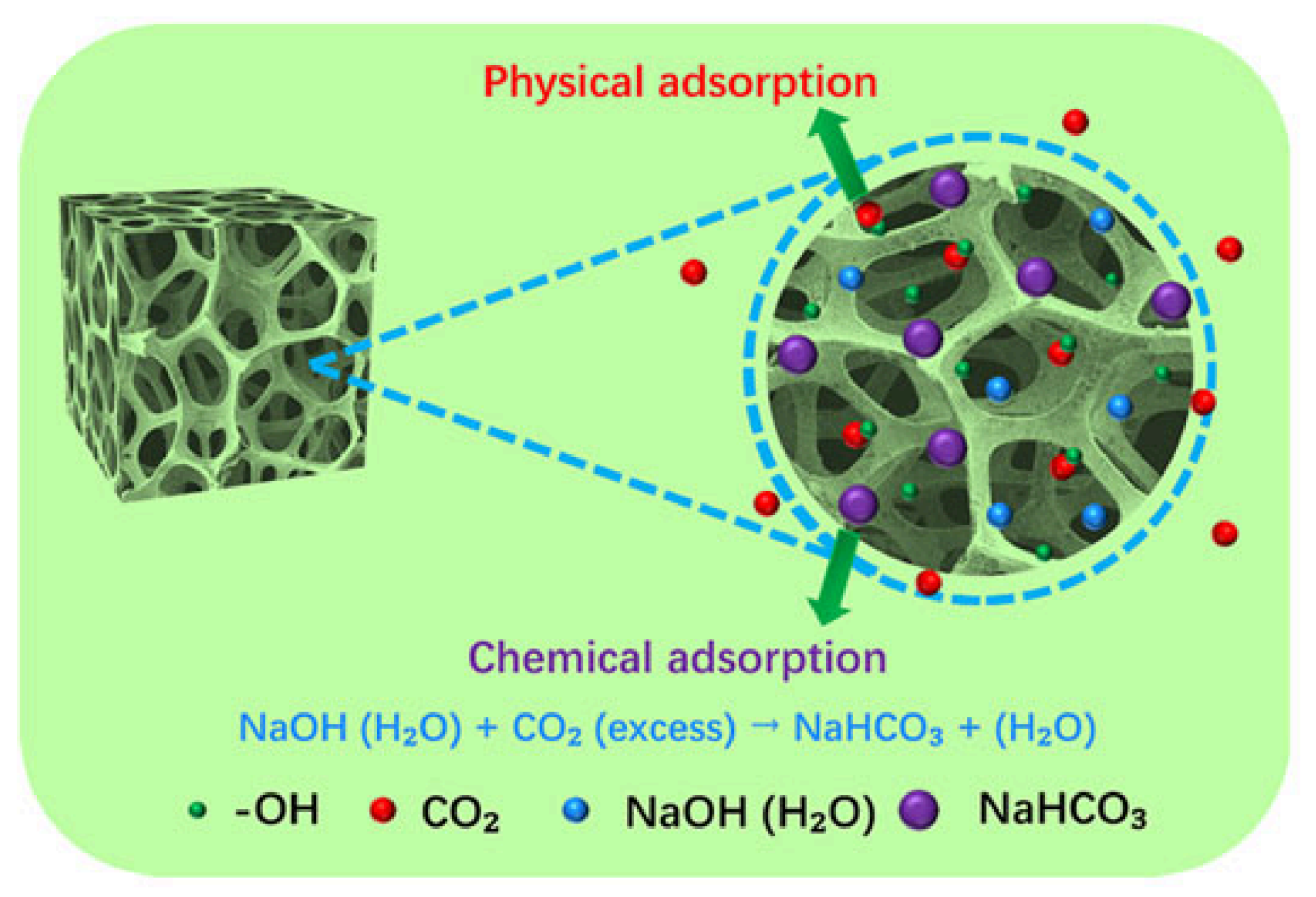
Schematic illustration of the physical and chemical adsorption processes of CO_2_ on aero-gels [95]. Copyright 2026, Elsevier.

Ji et al. [94] studied the amine-modified biochar–SiO_2_ hybrid aerogel prepared by supercritical drying technology under pure CO_2_ gas or binary mixed gas (such as CO_2_/N_2_ and CO_2_/Ar) conditions, utilizing the synergistic effects of physical adsorption with a three-dimensional porous structure, high specific surface area and ordered layered structure and chemical adsorption of amines. This material exhibits a CO_2_ adsorption capacity as high as 2.81 mmol/g at 0 °C and 1 bar, and it has a fast adsorption rate, reaching 90% of the equilibrium adsorption capacity within 17 min. In addition, this aerogel exhibits a high selectivity of 13–26 for CO_2_/N_2_ and can still maintain 80% of its initial adsorption capacity after four cycles, demonstrating excellent stability and regeneration performance. These characteristics give it great application potential in the field of post-combustion flue gas capture. Liu et al. [95] successfully prepared a hierarchical porous cellulose aerogel by the sol–gel method combined with freeze–thaw cycles and freeze-drying technology. The schematic diagram of the carbon dioxide adsorption process is shown in Figure 11. Micropores of 1–3 nm provide potential energy wells for physical adsorption, while macropores serve as “highways” for gas diffusion, significantly shortening the mass transfer path. Under dynamic conditions of simulated cement plant flue gas (CO_2_/O_2_/N_2_ = 10/20/70) at 25 °C and a flow rate of 10 mL/min, the breakthrough capacity reached 3.5 mmol/g. The aerogel exhibited good CO_2_ selectivity, and after multiple adsorption–desorption cycles, although the capacity decreased after the first cycle due to chemisorbent consumption, subsequent cycles maintained a stable adsorption capacity of approximately 1.0 mmol·g^−1^, indicating good reusability. Sami et al. [96] developed a novel amine-impregnated polymer aerogel for the efficient capture of CO_2_ from post-combustion flue gas. The large pore size facilitated the uniform dispersion of PEI at a loading of 4.1 g/g, avoiding the “sticky surface” phenomenon caused by pore blockage, thus achieving a high CO_2_ adsorption capacity of 4.45 mmol/g at 30 °C. Meanwhile, the material exhibited excellent regeneration stability, with the adsorption capacity remaining at 4.25 mmol/g after 5 cycles, corresponding to a retention rate of 95.5%, and complete regeneration could be accomplished in only 30 min at 120 °C, significantly reducing the energy demand of the post-combustion capture process.

In the field of post-combustion flue gas capture, the complex components in flue gas such as sulfur oxides, nitrogen oxides, oxygen, and water vapor have comprehensive effects on the stability and adsorption response of the materials. Although under dynamic conditions containing approximately 20% O_2_, oxygen and nitrogen can sometimes assist CO_2_ molecules in aggregating toward highly active sites, increasing the breakthrough capacity of the aerogel from 2.5 mmol/g to 3.5 mmol/g, long-term exposure still faces the risk of oxidative degradation of amine functional groups. More critically, acidic pollutants such as SO_2_ and NO_x_ can undergo irreversible reactions with chemisorption sites, leading to deactivation of active sites, pore blockage, and decreased mechanical strength. Therefore, in practical applications, pretreatment processes must be incorporated to extend the adsorbent lifespan. In addition, a rise in flue gas temperature (40–80 °C) leads to a decrease in exothermic adsorption capacity, while excessive humidity may induce capillary condensation that damages the nano-skeleton. By introducing stabilizers such as Ti(OH)_4_ to strengthen the hydrogen-bond network, the adsorption retention rate of the material in humid hot flue gas can be increased to above 85%, and rapid thermal regeneration at 120 °C can be achieved.

### 4.3. Natural Gas and Biogas Purification and Separation

The core challenge in CO_2_/CH_4_ separation for natural gas and biogas purification lies in the close kinetic diameters of the two molecules, requiring separation based on differences in adsorption affinity (thermodynamic selectivity) or molecular sieving effects (kinetic selectivity) [97,98,99].

CO_2_ possesses a quadrupole moment, while CH_4_ does not, which provides a thermodynamic basis for achieving selective adsorption through electrostatic interactions. Huang et al. [100] utilized carboxyl functional groups in biomass as bridges to grow Mg/Co-MOF-74 in situ, constructing a microporous/mesoporous synergistic structure. The micropore volume of the MOF increased from 0.02 cm^3^/g to 0.59 cm^3^/g, forming a deep potential well adsorption field, and by utilizing the high polarizability of CO_2_ molecules and the resulting strong electrostatic interactions, a high capacity of 6.4 mmol/g and extremely high selectivity were achieved. Meanwhile, the smaller kinetic diameter of CO_2_ compared with CH_4_ gives it lower diffusion resistance when passing through the hierarchical pores of the material, while the unique mesoporous and macroporous structures of the composite aerogel serve as “high-speed channels” to accelerate the transport of CO_2_ toward the MOF micropores.

Zhu et al. [101] investigated amino-functionalized nanocellulose aerogels (a-CNF aerogels) prepared via freeze-drying. These aerogels selectively adsorb CO_2_ over CH_4_ because CO_2_ adsorption primarily depends on chemisorption at amine active sites, whereas CH_4_ adsorption relies mainly on physisorption within the porous structure. The separation factor (α) of the aerogel for CO_2_/CH_4_ mixtures was greater than 1, indicating its ability to effectively separate CO_2_ and CH_4_. Atzori et al. [102] prepared two novel composite monolithic aerogels, sPS/UTSA-16/CHCl_3_ and PPO/UTSA-16/MB, via supercritical CO_2_ extraction. Their CO_2_ adsorption capacities reached 3.05 mmol·g^−1^ and 2.80 mmol·g^−1^, respectively. Meanwhile, these materials exhibited low affinity for CH_4_, with CH_4_ uptake far below that of CO_2_. At 298 K and 1 bar total pressure, for a 20:80 CO_2_/CH_4_ gas mixture, the selectivity factors of the sPS/UTSA-16/CHCl_3_ and PPO/UTSA-16/MB composite aerogels were 22.4 and 20.1, respectively, indicating their high efficiency in CO_2_ separation.

At present, the main purification and separation method for natural gas and biogas is the efficient separation of CO_2_/CH_4_. Aerogel has a high porosity and specific surface area. After being modified by amino functionalization or loading MOFs, it can provide a large number of chemical and physical adsorption sites, thereby achieving excellent CO_2_ adsorption capacity and high separation selectivity. However, its drawbacks are also quite obvious. The original aerogel usually has strong hydrophilicity and is prone to pore structure collapse in flue gas containing water vapor. Therefore, complex chemical modification must be carried out to enhance hydrophobicity. In addition, the preparation process of aerogel is energy-consuming and time-consuming. Moreover, while maintaining high adsorption activity, how to further enhance its mechanical strength to meet the self-supporting requirements of industrial adsorption towers remains the main challenge currently faced.

### 4.4. Carbon Sequestration

Carbon sequestration refers to the dynamic process of removing CO_2_ from the atmosphere over the long term and isolating it in stable reservoirs [103], i.e., capturing CO_2_ from the atmosphere and converting it into stable inorganic solid salts to achieve long-term environmental emission reduction [104], rather than merely temporary gas storage. Aerogels, by virtue of their unique nanoporous structures and tunable surface chemistry, efficiently capture and sequester CO_2_ through both physical and chemical adsorption mechanisms [105].

**Figure 12:**
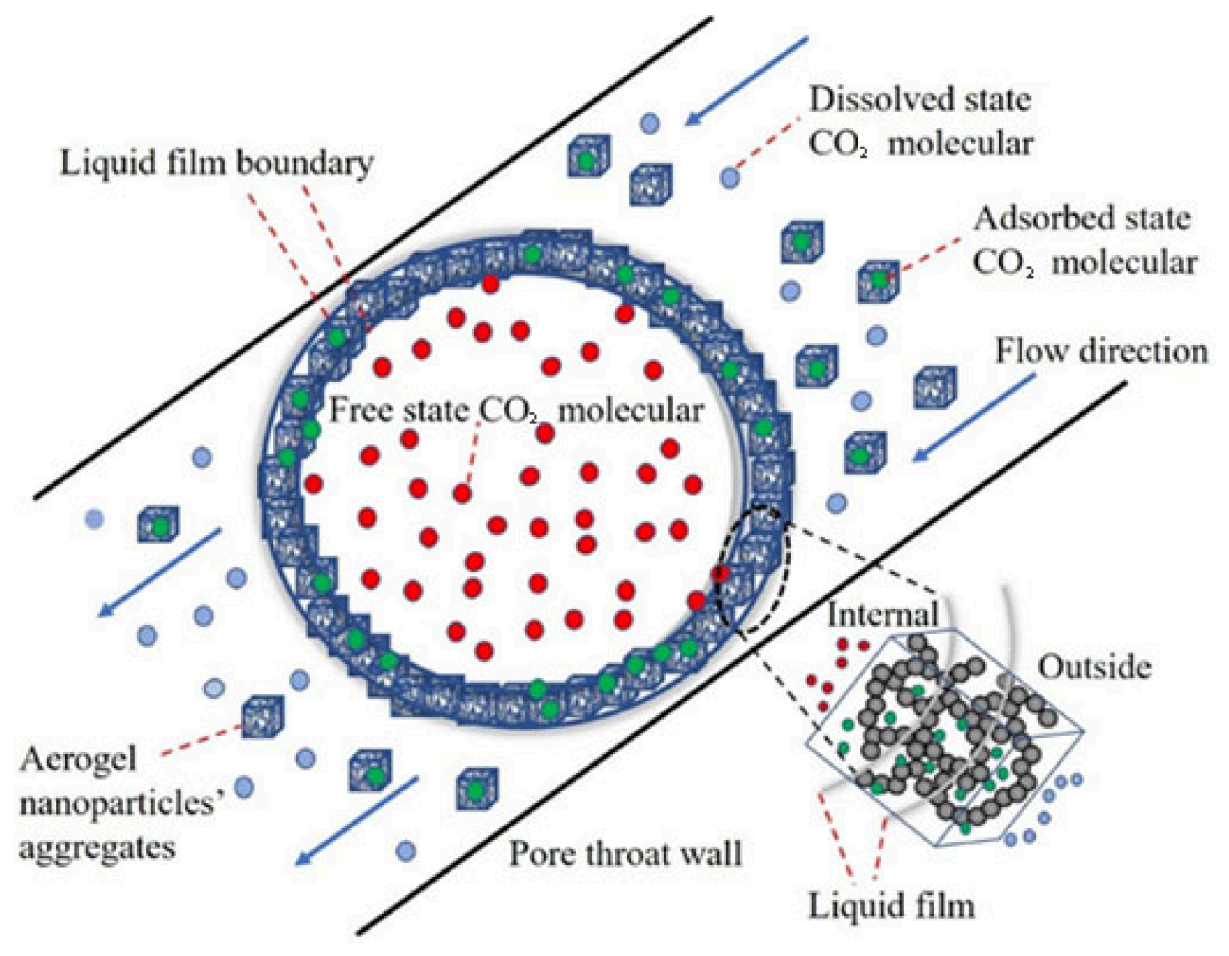
Different state models of CO_2_ in aerogels [106]. Copyright 2022, Elsevier.

Du et al. [106] used hydrophilic silica aerogel nanoparticles as stabilizers to prepare aerogel nanoparticle-stabilized foams via mechanical stirring. The aerogel nanoparticles aggregated at the gas–liquid interface, forming robust Pickering films that significantly enhanced foam stability. The foam increased the apparent viscosity of CO_2_ in porous media, thereby reducing its mobility and forcing CO_2_ into smaller pores. Through the blocking effect of the foam, CO_2_ was “locked” in the pore structure of the porous medium in the form of tiny bubbles, as shown in Figure 12, achieving physical sequestration and increasing CO_2_ storage capacity by approximately 30–50%. Lu et al. [107] utilized SiO_2_ aerogel nanoparticles to stabilize a flue gas foam system, successfully achieving efficient CO_2_ sequestration with a CO_2_ capture efficiency of 81.23%. At 95 °C, the gas diffusion rate of the aerogel-stabilized foam was five times lower than that of the pure surfactant foam, effectively inhibiting coalescence and coarsening of CO_2_ foam, thereby ensuring stable CO_2_ storage. Wang et al. [108] prepared cement aerogels via freeze-drying. The Ca^2+^ functional groups abundant in the cement aerogel, in synergy with the porous structure, converted CO_2_ into calcium carbonate solids through in situ mineralization, achieving a carbon fixation efficiency of 27.5%. This “mineralization” process permanently fixed gaseous carbon dioxide into the solid skeleton of the aerogel, making it a part of the material structure.

Aerogel materials not only serve as effective CO_2_ sequestration carriers but also significantly enhance sequestration stability, achieving efficient carbon storage. However, the synthesis of high-performance aerogels involves complex microstructure control and specific drying techniques, which increase the difficulty and technical threshold of industrial-scale production. Currently, the production cost of aerogel nanoparticles is relatively high. In carbon sequestration projects requiring large-scale injection, material costs and the loss of nanoparticles during recycling in complex subsurface environments remain major bottlenecks limiting widespread commercialization.

Based on the above discussion of various aerogel materials applied to CO_2_ capture in different scenarios, we have summarized the performance comparison of different aerogel materials in this field as shown in Table 4. Table 4 provides the detailed experimental conditions for the adsorption capacities, but these values may not be directly comparable across different conditions.

## 5. Regeneration and Desorption of Aerogels

The regeneration of aerogels is usually achieved through temperature increases (thermal desorption), pressure reductions (pressure swing adsorption), or a combination of both. Thermal desorption is the most common regeneration method, and its energy consumption mainly depends on the desorption temperature and the heat capacity of the adsorbent. The hydrophobic amine-functionalized silica aerogel has a relatively low regeneration temperature of only 65 °C under DAC conditions, which significantly reduces industrial operating costs. The PEI-impregnated polymer aerogel can complete regeneration in only 30 min at 120 °C, greatly reducing the energy demand of the post-combustion capture process. However, for certain adsorbents, excessively high regeneration temperatures may cause structural damage to the material or degradation of active sites, thereby affecting its cycle life.

Repeated adsorption–desorption cycles pose serious challenges to the mechanical strength and structural integrity of aerogels. The performance of aerogels significantly declines after multiple adsorption–desorption cycles, especially in oxygen-containing high-temperature flue gas, where amine groups are prone to oxidative deactivation. The fragile nano-skeleton of aerogels is susceptible to capillary condensation in humid air, leading to structural collapse. Therefore, future research should focus on developing aerogel materials with higher mechanical strength and stronger resistance to degradation, while the development of low-energy-consumption and mild regeneration strategies is an important direction for the commercialization of aerogel-based CO_2_ capture technology.

## 6. Conclusions and Prospects

This review comprehensively summarizes the three major preparation methods for aerogels, namely supercritical drying, ambient-pressure drying, and freeze-drying. It systematically classifies and reviews the design strategies for organic, inorganic, carbon-based, and composite aerogels, and thoroughly discusses the structure–performance relationships among amine functionalization, pore structure regulation, and CO_2_ adsorption performance. On this basis, the adsorption mechanisms and performance of aerogels in four typical application scenarios—direct air capture, post-combustion flue gas capture, natural gas and biogas purification and separation, and carbon sequestration—are summarized. Although aerogels exhibit significant advantages in the field of CO_2_ capture, numerous challenges remain on the path from laboratory research to practical engineering applications. Future research can be explored and improved in the following aspects:

(1) Aerogels exhibit unique advantages in CO_2_ capture compared with traditional adsorbents such as amine-modified mesoporous silica, activated carbon, zeolites, and MOFs. Their hierarchical pore structures can effectively shorten mass-transfer pathways and improve adsorption kinetics. However, traditional adsorbents also have their respective limitations, such as amine leaching, small differences in adsorption affinity, capacity decay under humid and hot conditions, insufficient hydrothermal stability, and high synthesis costs. Aerogels need to be further optimized in these aspects to stand out in the competition and provide more efficient and stable carbon capture solutions.
(2) The technological readiness of aerogels is still under development, with issues such as mechanical fragility and insufficient stability under humid and impurity-laden conditions. At the same time, current research is mostly limited to laboratory-simulated gas conditions, lacking systematic verification at pilot scale and under real gas conditions. To enhance long-term cycling stability, improve amine degradation resistance, optimize preparation processes to overcome problems such as ice crystal damage, and strengthen resistance to moisture and impurities for low-concentration DAC and complex flue gas capture scenarios, it is also necessary to advance long-term operational testing under real working conditions and pilot-scale demonstration to accelerate the industrialization of aerogel-based CO_2_ adsorption.
(3) The renewability and biodegradability of bio-based aerogels demonstrate their potential for environmental friendliness. However, a comprehensive life cycle assessment of aerogels is not yet complete. Future research should conduct in-depth analysis of the environmental impacts of aerogels throughout their entire life cycle, from raw material acquisition, production and manufacturing, use process, to final disposal, including energy consumption, greenhouse gas emissions, resource consumption, and waste generation. Through quantitative assessment, environmental hotspots can be identified to guide material design and process optimization, ensuring the overall sustainability of aerogels as carbon capture materials.
(4) The synthesis process of high-performance aerogels is complex, involving precise microstructure control and specific drying techniques, resulting in relatively high production costs. Future research should focus on developing more cost-effective preparation technologies, such as improved ambient-pressure drying processes, to reduce equipment investment and operating costs, so as to achieve low-cost, large-scale production of aerogels and thereby enhance their market competitiveness.

## Figures and Tables

**Table 1:** Comparison of major solid CO_2_ adsorbents with aerogels.

Material	Core Advantages	Key Limitations	Remaining Challenges	References
Amine-modified mesoporous silica	Chemical adsorption affords high selectivity; Ordered mesopores facilitate uniform amine dispersion; Suitable for moderate-temperature operation (60–80 °C)	Amine leaching causes continuous capacity decay; Mesopore blockage at high loading restricts mass transfer; Oxidative degradation (in O_2_/SO_2_ environments)	Improve thermal stability and oxidation resistance of amines during long-term operation; Reduce regeneration energy consumption	[4]
Activated carbon	Low cost; Abundant raw materials; High specific surface area (>1000 m^2^/g); Easy physical adsorption and regeneration	Low selectivity due to small difference in adsorption affinity between CO_2_ and N_2_; Insufficient capacity at low partial pressures	Significantly enhance selectivity without sacrificing capacity; Stability in humid environments	[5]
Zeolites	Low cost; Mature industrial-scale applications; Regular micropores provide a molecular sieving effect	Sharp capacity decline under humid conditions; High regeneration temperature (>300 °C)	Develop hydrophobic zeolites or composite structures to address moisture sensitivity; Reduce regeneration energy	[6,7]
Metal–organic frameworks (MOFs)	Highest structural design flexibility; Precisely tunable pore size and chemical environment; High theoretical capacity; Excellent selectivity	Insufficient hydrothermal stability; High synthesis cost; Difficult to shape in powder form	Scale-up production for cost reduction; Improve hydrothermal/chemical stability	[8]
Aerogels	Hierarchical pores (coexistence of micropores, mesopores, and macropores); Shorter mass-transfer pathways; Fast adsorption kinetics; Adaptable to various scenarios via amine grafting, N-doping, or MOF loading; Biomass-derived routes balance environmental friendliness and performance; Low regeneration energy; Support photothermal/electrothermal novel strategies	Mechanically fragile; Prone to fracture during industrial operation; Complex supercritical drying process; High cost; Pore blockage at high amine loading	Optimize ambient-pressure drying for scalable production; Verify long-term cycling stability (>1000 cycles); Enhance capacity for low-concentration DAC scenarios	[9]

**Table 2:** Comparison of preparation methods for aerogels.

Preparation Method	Effect on Gel Structure	Advantages	Disadvantages/ Challenges	Limitations	Cost	Scalability	References
Supercritical CO_2_ drying	Preserves the original nanoporous structure of the gel. Prevents shrinkage and collapse. Yields aerogels with high porosity and high specific surface area	Produces aerogels with high porosity, high specific surface area, and low density. Environmentally friendly	High equipment investment. Complex operation. Requires high-pressure equipment and precise temperature–pressure control. Energy-intensive; high operating costs. Time-consuming process	High investment in high-pressure equipment. Batch-type production limits industrial scale-up	High (due to high-pressure equipment investment)	Constrained by batch-type production mode, difficult for industrial scale-up	[14,15,16,20,21,22,23]
Ambient pressure drying	May cause collapse and pulverization of the gel network. Requires effective surface modification (e.g., hydrophobic modification) and internal structural reinforcement (e.g., crosslinking)	Low production cost Promising for large-scale industrial production Avoids the complexity of high-pressure or low-temperature operations	Gel prone to collapse; requires complex pretreatment. Difficult to achieve the low density and high porosity comparable to Supercritical drying	Prone to gel network shrinkage, collapse, and even pulverization; Requires complex chemical modification or strengthening strategies, which may sacrifice specific surface area or increase density	Low (low energy consumption, no need for extreme temperature/pressure equipment)	Easily adaptable to large-scale continuous production	[27,29,30,31,33,34,35]
Freeze drying	May cause aggregation of nanofibrils or nanoparticles Forms lamellar structures May introduce microcracks and loss of mesopore structure Can form layered pore structures	Avoids direct damage to the gel skeleton by liquid–gas interfacial tension. Relatively simple; no high-pressure equipment required	May cause physical damage to the gel structure. May result in relatively low mechanical strength. High cost. Relatively low efficiency. May cause aggregation of nanofibrils or nanoparticles	Traditional methods suffer from poor structural integrity, low mechanical strength, ice crystal damage and structural cracking; Relatively low specific surface area; Long drying cycles	Relatively high (long drying cycles and relatively high energy consumption)	Long drying cycles limit efficiency for large-scale production	[36,38,39,40]

**Table 3:** Comparison of different materials for composite aerogels.

Aerogel Type	Enhanced Property	Performance Enhancement Data	References
MOF	Mass transfer efficiency	Construction of a hierarchical “macro–meso–micro” pore architecture enhances mass transfer.	[66]
	Mechanical strength	The composite aerogel exhibits high mechanical strength.	[66]
	Dynamic CO_2_ separation productivity	CALF-20: 2.32 mmol·g^−1^; Zn-mtrz-ox: 1.70 mmol·g^−1^. The aerogel form shows approximately 45% improvement over the powder form.	[66]
	Adsorption efficiency for negatively charged organic pollutants	When CO_2_ concentration increases from 0 to 2000 mL·L^−1^, the adsorption efficiency increases from 39.47% to 74.57%.	[67]
	CO_2_ adsorption capacity	MOF@CNFs/DESL30G aerogel with 30 wt% DESL: CO_2_ adsorption capacity increased by 102.83%.	[68]
	CO_2_/CH_4_ selectivity	Selectivity coefficient of 7.5.	[68]
	Cycling stability	Retains 70% of its CO_2_ adsorption capacity after 6 adsorption cycles.	[68]
Ti_3_C_2_T_x_	Enzyme activity recovery	81.4%.	[72]
	Formic acid yield from CO_2_ reduction	Reaches 12.4% (based on 2 mM NAD^+^) in the photo-enzyme coupled system (PECS).	[72]
	Photocatalytic CO_2_ reduction efficiency	Total electron consumption rate of 112.6 μmol·g^−1^·h^−1^, which is 6.6 times higher than that of pristine CsPbBr_3_ NCs.	[73]
	Thermal conductivity	composite aerogel: 32.6 mW·m^−1^·K^−1^.	[74]
	Thermal insulation	After heating on a 180 °C hot plate for 5 min, the surface temperature of the composite aerogel is only 40.6 °C.	[74]
Polyethyleneimine	CO_2_ capture capacity	Up to 0.72 mmol·g^−1^.	[78]
	Amine efficiency	0.29.	[78]
	CO_2_ adsorption capacity	4.83 mmol·g^−1^ at 30 °C.	[79]
	Cycling stability	Retains 98.3% of its original capacity after 5 adsorption–desorption cycles; retains 92% stability after 6 cycles.	[78,79]
	Capture capacity under ambient CO_2_ concentration	Adsorption capacity of 0.61 mmol CO_2_·g^−1^ at 20 °C and 400 ppm CO_2_ partial pressure.	[80]
	CO_2_ adsorption capacity under high temperature and pressure	Adsorption capacity of 1.55 mmol CO_2_·g^−1^ at 50 °C and 101 kPa.	[80]

**Table 4:** Performance comparison of aerogels made from different materials.

Material Type	Design Strategy	Core Mechanism	Advantages	Limitations	Adsorption Capacity (mmol/g)	Working Conditions	Regeneration/Thermal Stability	Cyclic Stability and Applicability	References
Organic aerogels	Bio-based skeleton + amine modification	Utilizes abundant hydroxyl groups of cellulose/chitosan for crosslinking, and introduces PEI to enhance chemisorption.	Wide availability of raw materials, good biodegradability, low preparation cost.	Insufficient thermal stability, difficult to apply in high-temperature industrial flue gas.	9.26 mmol/g (100% CO_2_, 25 °C, static conditions) 2.44 mmol/g (1 bar, 25 °C, dynamic flow rate not specified) 3.5 mmol/g (simulated flue gas with 10% CO_2_, 25 °C, breakthrough capacity at 10 mL/min)	Ambient/low-temperature freeze-drying preparation	<300 °C	Maintains 7.13 mmol/g after 10 cycles; Good biodegradability	[40,41,42,104]
Inorganic aerogels	Si–O–Si rigid skeleton + hydrophobization	Prevents collapse via aromatic amine structures, combined with Ti(OH)_4_ to reinforce the hydrogen-bond network.	Excellent thermal stability and mechanical strength, supports low-energy regeneration.	High equipment investment (supercritical drying), complex modification process.	0.97–1.14 mmol/g (DAC conditions with 400 ppm CO_2_, 25 °C, 500 mL/min) 2.0 mmol/g (low-pressure mixed gas, 0.1–1 bar, 50 °C) 4.0 mmol/g (1 bar, 25 °C)	50 °C	>500 °C	Capacity retention >85% in humid flue gas; Relatively high cost	[45,46,47,88]
Carbon-based aerogels	N-doped chemisorption + micropore filling	Derived from carbonization of organic precursors, featuring conductive three-dimensional ordered honeycomb channels.	Enables photothermal/electrothermal regeneration strategies, good mechanical elasticity.	Poor selectivity in physisorption; pores tend to be blocked after modification.	4.88 mmol/g (100% CO_2_, 25 °C, oriented structure) 2.6 mmol/g (1 bar, 25 °C, N-doped) 2.81 mmol/g (1 bar, 0 °C, hybrid aerogel)	High-temperature carbonization preparation	Good chemical stability	Supports Joule heating regeneration; Good mechanical elasticity	[58,59,60]
Composite aerogels	Hierarchical interconnected pores + electrostatic interaction	Utilizes electrostatic attraction between open metal sites and the CO_2_ quadrupole moment, constructing “macro–meso–micro” mass-transfer channels.	Extremely high theoretical capacity ceiling, excellent selectivity (separation factor up to 594).	High-cost, difficult-to-shape crystalline powders.	6.4 mmol/g (100 kPa/1 bar CO_2_, 298 K) 3.15 mmol/g (low-pressure flue gas at 0.15 bar, 298 K) 8.4 mmol/g (1 bar CO_2_, 273 K/0 °C)	Performs excellently in CO_2_/CH_4_ and CO_2_/N_2_ mixtures. Conducted at 1 bar	Depends on substrate, generally stable	Retains 96.8% of its initial capacity after 10 cycles.	[66,67,68,72,73,74,78,79,80,98,105]
	2D functionalization + interfacial hydrogen-bond regulation	Exploits the high conductivity of Ti_3_C_2_T_x_ and surface polar groups (–O, –OH) to enhance CO_2_ affinity.	Imparts unique photocatalytic activity and electrochemical functional sites to the material.	Risk of particle loss, sensitivity to interfacial defects.	1.55 mmol/g (101 kPa, 50 °C, dynamic conditions) 0.72 mmol/g (1 bar, 30 °C) 0.61 mmol/g (DAC conditions with 400 ppm CO_2_, 20 °C)	The adsorption efficiency in gas–liquid–solid three-phase systems is notably influenced by the flow rate. It is commonly used in photo-enzyme coupled reduction.		Retains 70% of its capacity after 6 cycles.	
	Multi-level amine groups + covalent capture	PEI acts as both a chemical crosslinker and capture agent, forming carbamates to fix the gas.	Extremely strong capture capacity at low concentration (400 ppm), supports moisture-swing regeneration.	Risk of amine volatilization; trade-off between regeneration temperature and energy consumption.	4.45–4.83 mmol/g (50% CO_2_, 1 bar, 30 °C) 3.71 mmol/g (DAC conditions with 400 ppm CO_2_, 25 °C, high-humidity MSQCA) 0.72 mmol/g (DAC conditions with 400 ppm CO_2_, 25 °C, dry environment)	The capacity can be significantly enhanced under high-humidity conditions. Desorption is completed within 30 min at 120 °C.		It still exhibits excellent mechanical robustness after 30 wet–dry cycles.	

## Data Availability

No new data were created or analyzed in this study. Data sharing is not applicable to this article.
